# A two-year perspective: who may ease the burden of girls’ loneliness in school?

**DOI:** 10.1186/1753-2000-8-10

**Published:** 2014-04-08

**Authors:** Audhild Løhre, Marianne N Kvande, Odin Hjemdal, Monica Lillefjell

**Affiliations:** 1 Research Centre for Health Promotion and Resources, Trondheim, Norway; 2 Department of Social Work and Health Sciences, Norwegian University of Science and Technology, Trondheim, Norway; 3 Department of Psychology, Norwegian University of Science and Technology, Trondheim, Norway; 4 Department of Occupational Therapy, Sør-Trøndelag University College, Trondheim, Norway

## Abstract

**Background:**

Loneliness is negatively related to good health and wellbeing, especially among girls. There is little research, however, on factors that may ease the burdens of loneliness in the school setting. Thus, we explored the relationship between girls’ loneliness and later school wellbeing adjusted for other adversities. Furthermore, we assessed the significance of having someone whom the girl trusted by investigating possible modifying influences on the addressed association.

**Methods:**

Altogether, 119 girls in grades 1–8 provided baseline data and answered the same set of questions two years later. Logistic regression models including perceived academic problems, victimisation by bullying, loneliness and trusted others were tested with bad versus good school wellbeing two years later as outcome using SPSS.

**Results:**

In the multivariable analysis of loneliness, academic problems, and victimisation, loneliness was the only variable showing a strong and negative contribution to later school wellbeing. Next, demonstrated in separate models; the inclusion of having a trusted class advisor fully attenuated the association of loneliness with later school wellbeing. In contrast, other trusted teachers, trusted parents, or trusted students did not affect the association.

**Conclusions:**

Loneliness in girls strongly predicted school wellbeing two years later. However, having a class advisor whom the girl trusted to contact in hurtful situations clearly reduced the burden of loneliness. This finding highlights the clinical importance of stability, long-lasting relations, and trust that main teachers may represent for lonely girls.

## Background

Loneliness is a threat to students’ wellbeing and health. Perceived loneliness at school has shown negative associations with both emotional and somatic symptoms, especially among girls [1]. Furthermore, loneliness in girls has demonstrated longitudinal associations with lower levels of perceived school wellbeing [2]. Thus, research on loneliness indicates an urgent need to search for factors that may reduce the burden of girls’ loneliness. Other adversities related to loneliness are victimisation, caused by bullying, and the perception of having academic problems. It has been shown that students with low levels of academic achievement or learning disabilities are lonelier, have poorer social adjustment and more emotional problems than students with average or high levels of academic achievement [3-6].

The subjective experience of being victimised by bullying has often been measured by the frequency of verbal and/or physical harassment and also by the frequency of social exclusion [7]. Definitions of bullying usually include imbalance of power; aggressive behaviour; and repetitive negative acts [8,9]. The prevalence of victimisation has been shown to vary considerably between countries, even when the same measurements have been applied [10,11]. There seems, however, to be a consensus that victimisation is related both to mental health problems [12]; poor psychosocial adjustment [13-15]; and to a higher risk of psychosomatic problems [16,17]. The association between victimisation and ill health has been found to be fairly similar between countries [18]. Further, a dose–response relation between victimisation and ill health has been reported, in that an increased frequency of victimisation was related to higher levels of ill health symptoms [10,19,20].

Loneliness is less studied than victimisation and has received far less public attention during the last decades. As summarised by Peplau and Perlman [21], definitions on loneliness typically comprise an unpleasant or distressing subjective experience of deficiencies in a person’s social relationships. The distinction between loneliness and aloneness is crucial [22]; aloneness may give time for reflection and rest, whereas loneliness is a negative and hurtful feeling [23,24]. In line with the findings in research on victimisation, it has been shown that loneliness is related to mental health and adjustment problems [25]. There is strong evidence that loneliness is related to anxiety [26-28] and depression [29-31], but few studies have reported associations between loneliness and somatic symptoms [1]. Loneliness among children and adolescents has been studied mainly in the school setting, and few researchers have tested initiatives to buffer feelings of loneliness [32-34]. Furthermore, as far as we are aware, no studies have reported on relational trust as a potential to reduce harmful effects of loneliness in school.

Resilience is a research approach that focuses on factors and processes buffering the effects of adversity and stressful life events. Consistently across studies, growing up with at least one trusted person has been identified as a very important protective factor. This could be a parent or another person, such as a teacher, coach, or neighbour [35-37]. It has also been found consistently throughout the resilience research that growing up with an early established and secure attachment to the caregiver is important for the development of a capacity to trust, and for the stimulation of emotional regulation and mentalising capacities (e.g. self-reflection) [35,38]. Having one adult who can be trusted, such as a parent, neighbour, trainer or a teacher may be an especially important protective factor in buffering adversity.

### Aims of the study

The first aim was to investigate the association of perceived loneliness with female students’ self-reported school wellbeing two years later in a model adjusting for perceived academic problems and victimisation caused by bullying. The second aim addressed the relationship between loneliness and later school wellbeing and assessed the influence of having a range of people whom the girls trusted sufficiently to contact if hurtful or difficult situations arose.

## Methods

### Participants and procedure

In this study, 119 girls from five convenience sampled public schools in Mid-Norway provided information two years apart; May-June 2002 (T1) and May-June 2004 (T2). At T1 the girls were in grades 1–8 (age 7–14) and at T2 in grades 3–10 (age 9–16). The total population of girls and boys at baseline and the transmission of students to other schools in the project period are described in detail elsewhere [2]. The rate of participation at the two-year follow-up was 99%.

Data were collected by using the School Wellbeing Questionnaire (SWQ) [39]. School nurses and headmasters administered the data collection. The younger students were interviewed by trained school nurses who used the questionnaire as a guide, whereas the older students completed the questionnaires themselves under the instruction of a trained teacher or the school nurse during a lesson allocated to this task. More information about the instrument and methods are available in other publications [1,39].

### Measures

The SWQ contained items on three potential areas of adversity: perceived academic problems; victimisation (being bullied); and loneliness. Furthermore, the students’ comments on having someone to turn to in difficult situations were included in the SWQ in addition to measures of the outcome of perceived school wellbeing.

#### *School wellbeing at T1 and T2*

One global question: “How do you like it at school?” with four response options; very bad (1), not so good, good, and very good (4).

#### *Academic problems at T1*

Four questions each linked to a certain subject: “Do you have problems with: reading”; “writing”; “mathematics”; or “foreign language (English)?” Each had four response options: no problems (1), some problems, quite a few problems, and lots of problems (4). In the analysis, we used the question(s) with the highest response score of the four questions (the maximum score, i.e. one score only).

#### *Victimisation at T1*

Three questions each prefaced by: “During recess, are you bothered in some way that makes you feel bad?” and the following then were specified: being “teased”; being “hit, kicked or pushed”; and being “left out, excluded”. Each of the three questions had five response options; never (1), seldom, sometimes, about every week, and about every day (5). The maximum score of the three questions was used in the analysis.

#### *Loneliness at T1*

One question: “Do you ever feel lonely at school?” with five response options; never (1), seldom, sometimes, about every week, and about every day (5).

#### *Trusted others at T1 and T2*

Five questions each linked to identified groups of people: “Who can you talk to if something hurtful or difficult happens to you: class advisor”; “other teachers”; “other students”; “your parents”; “other adults”? Each question had four response options; no-never (1), maybe, probably, and certainly (4).

### Statistics

The distribution of the population of 119 girls was described by the dispersion, median, and interquartile range (IQR) of the outcome (school wellbeing T2) and the independent variables. Correlations between trusted others at T1 and T2 were assessed by Spearman’s rho. For use in logistic regression analyses, the outcome was dichotomised into the categories of bad (very bad/not so good) and good (good/very good) school wellbeing. Associations between the potential adversities and the dichotomised outcomes were tested in a multivariable analysis, adjusting for grades and earlier school wellbeing. Next, 10 multivariable adjusted models were constructed by including each of the five groups of trusted people separately; firstly, using the scores at T1 and secondly, using the scores at both T1 and T2. The multivariable models were also calculated with adjustments made for schools. All tests were two-sided, and p-values <0.05 were considered significant. The statistical analyses were performed in SPSS for Windows (version 20.0 SPSS, Chicago, Illinois).

### Ethics and procedures

The surveys were approved by the statutory School Collaborative Committees, and the collection of data was approved by The Norwegian Data Inspectorate. Information letters signed by the headmaster and by the principal investigator (AL) were sent to all parents, describing the aims of the surveys, and emphasising that participation was voluntary, and that the collected information was confidential. In addition, parents were informed about the surveys in school meetings and, in each class, teachers informed in greater details. Students/parents who did not want to participate were asked to notify their main teacher or headmaster; however, no parent or student refused to take part.

## Results

Self-reported school wellbeing levels were high with more than 90% reporting good or very good (Table 1). Fewer than 10% reported quite a few or lots of academic problems; about 6% reported weekly or daily victimisation; and just over 3% experienced loneliness weekly or daily. Parents were the most trusted group of people with approximately 80% of the girls at T1 and 89% at T2 saying they would probably or certainly turn to them in difficult situations. Class advisors formed the second most trusted group of people at T1 and the third most trusted at T2, competing the group comprising other students. The greatest increase in trust was seen in the group consisting of other students: the percentage of girls reporting that they trusted other students increased from 58% at T1 to 74% at T2.

**Table 1:** Distribution of response options for the outcome and the independent variables

**Variables**	**Response options**					**Total**	**Median**	**IQR**^**#**^
	**1**	**2**	**3**	**4**	**5**			
	**%**	**%**	**%**	**%**	**%**			
School wellbeing T2^a^	3.4	3.4	42.4	50.8		118	4	3–4
School wellbeing T1^a^	0	6.8	47.0	46.2		117	3	3–4
Academic problems T1^b^	34.5	56.3	7.6	1.7		119	2	1–2
Victimisations T1^c^	50.4	24.4	19.3	3.4	2.5	119	1	1–3
Loneliness T1^c^	52.1	24.4	20.2	1.7	1.7	119	1	1–2
Class advisor T1^a^	16.0	17.9	16.0	50.0		106	3.5	2–4
Other teachers T1^a^	21.1	22.1	18.9	37.9		95	3	2–4
Students T1^a^	15.1	26.4	13.2	45.3		106	3	2–4
Parents T1^a^	3.6	16.2	8.1	72.1		111	4	3–4
Other adults T1^a^	26.7	36.0	17.4	19.8		86	2	1–3
Class advisor T2^a^	11.1	21.4	13.7	53.8		117	4	2–4
Other teachers T2^a^	17.5	28.1	21.9	32.5		114	3	2–4
Students T2^a^	0.9	25.0	21.6	52.6		116	4	2–4
Parents T2^a^	3.4	7.6	10.2	78.8		118	4	4–4
Other adults T2^a^	25.9	33.3	18.5	22.2		108	2	1–3

^#^25-75th percentile.

^a^From 1 (worst) to 4 (best).

^b^From 1 (best) to 4 (worst).

^c^From 1 (best) to 5 (worst).

Note: Loneliness at T1 was the variable of special interest with School wellbeing at T2 as the outcome. Adjustments included School wellbeing, Academic problems, and Victimisation; all at T1. People, whom the girls trusted to contact at T1, were assessed to see if any of those groups of persons modified the association of loneliness with later school wellbeing. Corresponding groups of persons at T2 were included as adjustment.

Correlations were calculated to assess any changes, between T1 and T2, in the degree of trust felt for specified groups of trusted others (Table 2). For class advisor, students, and parents, the correlations between T1 and T2 were statistically significant, but below rho 0.40. Other teachers and other adults showed non-significant correlations between the two points in time.

**Table 2:** Spearman’s rho correlations: trusted others

	**Class advisor T2**	**Other teachers T2**	**Students T2**	**Parents T2**	**Other adults T2**
Class advisor T1	0.39^**^	0.21^*^	0.01	0.08	-0.02
Other teachers T1	0.37^**^	**0.19**	0.05	0.29^**^	0.16
Students T1	0.17	0.08	0.37^**^	-0.03	0.14
Parents T1	0.22^*^	0.27^**^	0.07	0.25^**^	0.27^**^
Other adults T1	0.38^**^	0.19	0.12	0.16	**0.13**

^*^p < 0.05.

^**^p < 0.01.

Note: Correlations between T1 and T2 for the same groups of trusted others are marked with bold numbers.

Associations between the potential adversities at T1 and school wellbeing at T2 were explored by mean of a multivariable regression analysis, adjusted for grades and school wellbeing at T1 (Table 3). For lonely girls, the odds of reporting high levels of school wellbeing two years later were reduced by 65% compared to other girls (odds ratio, 0.35, 95% CI 0.13 to 0.92). Loneliness was the only variable demonstrating a strong and negative independent contribution.

**Table 3:** **Associations of potential adversities (T1) with school wellbeing (T2)**^**¤**^

***Adverse factors***	**Odds ratio (95% CI)**	**p-value**
Academic problems	0.89 (0.24 to 3.32)	0. 862
Victimisation	1.91 (0.68 to 5.40)	0. 223
Loneliness	0.35 (0.13 to 0.92)^*^	0. 033

^¤^adjusted for grades and school wellbeing (T1) in a multivariable logistic regression analysis.

^*^Note: The longitudinal association of loneliness with later school wellbeing was strongly negative, with p-value < 0.05.

Next, the influence was explored of each of the trusted others upon the relationship between loneliness at T1 and school wellbeing at T2. In Table 4, each of the five groups of trusted others was added separately (a-e) in the multivariable analyses, adjusted for grades and school wellbeing at T1. The question to be answered was whether any of the trusted others modified the negative association of loneliness with later school wellbeing. In Model 1a-e, the scores of trusted others at T1 were included, one by one. Further, to assess a possible influence of trusted others at T2, the scores of trusted others at T1 and T2 were included simultaneously in Model 2a-e. In Model 1a, the influence of loneliness on later school wellbeing was fully attenuated by having a trusted class advisor at T1. By adding class advisor at T2 (Model 2a), the association was even weaker, and additionally, class advisor at T2 demonstrated a strong and positive independent contribution (odds ratio, 3.68, 95% CI 1.06 to 12.79). Other adults at T1 showed a corresponding modifying influence on the association between loneliness and later school wellbeing (Model 1e), but this influence was somewhat reduced when other adults at T2 was added (Model 2e). None of the other groups of trusted persons (other teachers, students, or parents in Models b-d) affected the strongly negative association between loneliness and later school wellbeing, except other teachers at T1 and T2 when they were included simultaneously (Model 2b).

**Table 4:** Influence of trusted others on the relation between loneliness (T1) and school wellbeing (T2)

	**Model 1 (a-e)**^**¤**^		**Model 2 (a-e)**^**¤**^	
	**Odds ratio (95% CI)**	**p-value**	**Odds ratio (95% CI)**	**p-value**
**a. Class advisor**				
Loneliness	0.48 (0.16 to 1.41)^§^	0. 181	0.52 (0.16 to 1.68)^§^	0. 272
Class advisor T1	1.99 (0.79 to 5.01)	0. 145	1.51 (0.60 to 3.79)	0. 382
Class advisor T2			3.68 (1.06 to 12.79)	0. 040
**b. Other teachers**				
Loneliness	0.30 (0.10 to 0.88)	0. 029	0.35 (0.12 to 1.05)^§^	0.060
Other teachers T1	1.02 (0.46 to 2.25)	0. 963	1.10 (0.49 to 2.47)	0. 813
Other teachers T2			1.64 (0.67 to 3.99)	0. 279
**c. Students**				
Loneliness	0.35 (0.12 to 0.97)	0.043	0.25 (0.06 to 0.96)	0. 043
Students T1	1.66 (0.72 to 3.83)	0.239	1.20 (0.45 to 3.16)	0. 720
Students T2			2.79 (0.78 to 9.95)	0. 115
**d. Parents**				
Loneliness	0.26 (0.08 to 0.81)	0. 021	0.24 (0.07 to 0.78)	0. 017
Parents T1	0.54 (0.15 to 1.91)	0. 339	0.58 (0.17 to 2.03)	0. 396
Parents T2			0.74 (0.24 to 2.24)	0. 589
**e. Other adults**				
Loneliness	0.50 (0.17 to 1.49)^§^	0. 212	0.38 (0.12 to 1.27)^§^	0.117
Other adults T1	1.13 (0.41 to 3.09)	0. 813	1.29 (0.46 to 3.61)	0. 629
Other adults T2			0.62 (0.25 to 1.55)	0. 307

^¤^adjusted for academic problems, victimisation, school wellbeing and grades (T1) in multivariable logistic regression models.

^§^the influence of loneliness turns to be non-significant (p-value ≥ 0.05).

Note: In Model 1 (a-e), the scores of each group of trusted others at T1 were included separately in Model 1a to Model 1e. In the right part of the table (Model 2 (a-e)), the scores of each group of the trusted others at T1 and T2 were included simultaneously.

Participants in this study were recruited from five schools, and it could be asked whether ‘school’ should have been included as a covariate in the adjustments, together with grade and school wellbeing at T1. Because of the relatively low number of participants, the covariates were kept to a minimum; therefore, school was not included in the results presented above. However, we ran corresponding analyses to those in Tables 3 and 4, also adjusting for school. When school was included, the negative association between loneliness and school wellbeing was even stronger (odds ratio, 0.27, 95% CI 0.09 to 0.76) compared to the corresponding association in Table 3. The influence of teachers was generally weaker when school was included but, in line with the tabulated results, class advisor still fully attenuated the association of loneliness with later school wellbeing. The influence of other teachers, and also other adults, disappeared compared to Models 2b and 2e, respectively. For students and parents, there were no substantial changes.

## Discussion

This longitudinal study assessed the influence of different groups of trusted people on the relationship between girls’ perceived loneliness at school and their self-reported school wellbeing two years later. Among the 119 girls in grades 1–8, loneliness at school was strongly related to low levels of school wellbeing. However, having class advisors whom they trusted sufficiently to turn to in difficult or hurtful situations fully attenuated the negative association of loneliness with later school wellbeing. Also, other non-specified adults fully attenuated the longitudinal association between loneliness and school wellbeing. On the other hand, trusted people such as parents, peers at school, or other teachers did not substantially affect the relationship between girls’ loneliness and later school wellbeing. For parents, this is especially surprising since 80-90% of the girls reported, two years apart, that they probably or certainly would talk to their parents if something bad or difficult happened.

### The influence of potential adversities

Of the three potential adversities; academic problems, victimisation and loneliness, the latter was the only adversity showing a strong relationship with later school wellbeing. It has been shown previously in cross-sectional studies that loneliness may be more damaging to wellbeing at school than both victimisation caused by bullying and students’ perceptions of academic problems [39-41]; however, this relationship has been inadequately explored in longitudinal studies [2].

### Stability of trust over the two years

For each group of trusted persons, the correlations between T1 and T2 were unexpectedly low. For parents especially a higher stability was expected between the two points in time as parents constituted by far the most trusted group at both data collection points. We did not find theoretical or empirical support for this low correlation of rho 0.25. The girls reported the highest stability of trust in the class advisor, a finding that might be ascribed to the important role of main teachers in Norwegian schools. Contrary to this, other teachers and other adults showed no significant stability. This may reflect that other teachers and other adults, such as coaches, change during a few school years.

### The importance of trusted teachers

Our results highlight the great importance of teachers to girls who feel lonely at school. Among lonely girls, who trusted their class advisor sufficiently to contact her in difficult or hurtful situations, statistical analysis showed that reported loneliness did not significantly affect their later wellbeing at school. This indicates that lonely girls were just as likely to experience good school wellbeing as non-lonely girls.

One possible explanation of the class advisor’s impact on the relationship between loneliness and wellbeing at school may be that these three measures are all related to the school setting. This cannot be the only explanation, however, as our data demonstrated a notable difference between the impact of the class teacher and that of other teachers. This difference may be related to the Norwegian school system where the class advisor represents stability by teaching most of the lessons, typically over 3–4 years, whereas other teachers meet the students less frequently. Besides being a buffer for the harmful influence of loneliness over the two year perspective, reports on trusted class advisor at T2 also showed a concurrent and strong association directly with the girls’ school wellbeing.

The results indicate that trust in other teachers was less important to the girls’ school wellbeing; other teachers had no influence on the negative association of loneliness with school wellbeing two years later and, by adding trust in other teachers at T2, the association was only modestly changed. In line with the resilience literature [42,43], there are reasons to suggest that stable and long-lasting relationships with main teachers are valuable to most students, and especially to those who feel lonely.

### The role of others

Our results demonstrated that parents were the most trusted group of people, and this corresponds to findings illustrating the importance of children and adolescents being closely attached to their parents [44]. The results revealed in the multivariable analyses therefore were surprising; having trust in parents did not ease the burden of loneliness; in fact, the negative association between loneliness and later school wellbeing was enforced by adding ‘trusted parents’. One explanation of this finding may be that parents are usually separated from the school setting, only occasionally taking part in school activities with their children and the teachers. The ‘setting’ argument is, however, questionable seen in light of the minimal influence of other teachers who, by definition, are inside the school setting. Another explanation could be a weak attachment [45] between the lonely girl and her parents; it is possible that lonely girls tend not to trust their parents. However, our study was not designed to answer that possibility. A third explanation may be that the lonely girls hid their hurtful feelings related to perceived loneliness at school and never told their parents, corresponding to hidden feelings of shame and inferiority linked to invisible symptoms of depression and anxiety [46,47]. Nevertheless, the suggestions above leave the question open: why do we not see any positive influence of trusted parents on the association of loneliness with later school wellbeing?

Positive peer relationships, such as having friends, being accepted by peers and the quality of friendship are shown to protect against loneliness [48,49]. We are not aware of any study that discusses the role of peers in buffering the harmful effects of loneliness. In our study, reporting trust in other students had no influence on the relationship between loneliness and later school wellbeing. Cassidy and Berlin suggested that adequate peer relations mediate a link between weak parent attachments and loneliness in children [45]. To our knowledge, no studies have addressed this hypothesis.

In accordance with findings in the resilience research [35,37], our results showed that other trusted adults may mitigate the bad influence of adversity, in this case loneliness. Those adults were probably outside the school setting, but are not identified in our data. They might have been a relative, a trainer, or someone else who was trusted – and maybe the only one [50,51].

### Strengths and limitations

The longitudinal population based design and the very high rate of participation are the strengths of this study. The schools were all public and ranged from inland to costal environments in rural communities. A weakness of the study is that students from urban settings were not included, and it is difficult to anticipate to what degree the results from this convenience sampling of schools may be generalised. Furthermore, it is possible that psychiatric co-morbidity (not included in the study) may have affected the results by influencing perceptions of loneliness; placing trust in other people; and perceptions of school wellbeing. Later studies should consider including measures of emotional symptoms or diseases. All students answered the same set of questions and were guided by school nurses or teachers, all of whom were trained and knew the purpose of the study. The younger students were interviewed by the nurses, whereas the older students completed their questionnaires in a lesson allocated to this task, guided by a teachers or a nurse. These different procedures could have introduced systematic errors between the younger and older student groups. Nevertheless, the congruence in the influence of trusted others between T1 and T2 indicate that the findings are robust and can withstand variations in methods, as well as in age, in this student population. It will, however, be of great value to replicate this study using larger samples or populations.

## Conclusions

Loneliness in girls strongly predicted school wellbeing two years later. Among three potential adversities, loneliness was the only variable showing a strong and negative longitudinal association with school wellbeing. The perception of having academic problems, or being victimised by bullying, did not contribute individually to school wellbeing. However, having a class advisor at T1 whom the girl trusted sufficiently to contact in stressful situations clearly reduced the burden of loneliness. Also, having another trusted adult (not identified) at T1 eased the burden of loneliness. In contrast, other trusted people at T1 such as trusted parents, students, or other teachers did not affect the relationship between loneliness and later school wellbeing. Furthermore, adjusting for the same groups of trusted others at T2 in the respective analyses showed no substantial changes in the results. This demonstrated the consistency in the results; the longitudinal associations were fairly similar to the cross-sectional associations. The influence of other adults should be recognised but, as they were not identified as individuals, they cannot guide interventions. On the contrary, the impact of the main teacher – the class advisor – calls for attention in schools, health services and public health in general. This finding highlights the great clinical importance of the stability and trust that main teachers may represent for their students, and especially for lonely and vulnerable girls.

### Consent

Data in this publication was drawn from surveys in a school project. All students and parents were given oral as well as written information about the surveys and the project. They were told that participation in the surveys was voluntary, and that the collected information was confidential. Students/parents who did not want to participate were to inform the headmaster or their class advisor. No parents or students denied participating, and informed consent was given by participating and completing questionnaires.

## Competing interests

The authors declare that they have no competing interests.

## Authors’ contributions

All four authors participated in the design, interpretation of data, and writing of the paper. AL and OH did the analyses. All authors read and approved the final manuscript.
